# Time Trend Analysis of Comorbidities in Ankylosing Spondylitis: A Population-Based Study from 53,142 Hospitalizations in Poland

**DOI:** 10.3390/jcm13020602

**Published:** 2024-01-21

**Authors:** Katarzyna Helon, Małgorzata Wisłowska, Krzysztof Kanecki, Paweł Goryński, Aneta Nitsch-Osuch, Krzysztof Bonek

**Affiliations:** 1 Department of Rheumatology, National Institute of Geriatrics, Rheumatology and Rehabilitation, 02-637 Warsaw, Poland; mwislowska@wp.pl (M.W.); krzysztof.bonek@spartanska.pl (K.B.); 2 National Institute of Public Health—National Institute of Hygiene, 00791 Warsaw, Poland; kanecki@mp.pl (K.K.); pawel@pzh.gov.pl (P.G.); aneta.nitsch-osuch@wum.edu.pl (A.N.-O.)

**Keywords:** ankylosing spondylitis, cardiovascular risk, epidemiology, atherosclerosis

## Abstract

Background: (1) Influence of comorbidities on life expectancy and treatment outcomes is one of the main concerns of modern rheumatology, due to their rising prevalence and increasing impact on mortality and disability. The main objective of our study was to analyze the time trends and shifts in the comorbidity profile and mortality over 10 years in the Polish population with ankylosing spondylitis (AS). (2) Data from 2011–2020 years were acquired from the General Hospital Morbidity Study in the National Institute of Public Health—National Institute of Hygiene (NIH-PIB) as ICD-10 codes. Based on ICD10 codes, we calculated the percentage shares for comorbidities, with the relative risk ratios and odds ratios. We analyzed the hospitalization rates and mortality from the overlapping conditions. Also, we analyzed age and sex related differences in the clinical manifestations of AS patients. (3) Results: From 53,142 hospitalizations of patients with AS, we found that the male population presented higher rates of cardiovascular (2.7% vs. 1.3% *p* < 0.001) and pulmonary conditions (1.2% vs. 0.8% *p* < 0.025). Inflammatory bowel diseases were more common in the female population than in males (2.3% vs. 1.7%, *p* < 0.001). In the years 2011–2020, we observed a decline in the number of hospitalized patients due to cardiovascular (*p* < 0.001) and respiratory system conditions (*p* < 0.001), yet the relative risk and odd ratios remained high. In the years 2011–2020, 4056 patients received biological treatment (7%). The number of initiated biological therapies correlated negatively with the number of reported hospitalizations due to ischemic heart diseases (IHD) (*p* < 0.031, r = −0.8). Furthermore, in the logistic regression model, we found strong collinearity between cardiovascular and pulmonary comorbidities (VIF = 14; tolerance = 0.1); also, the number of reported IHD’s correlated positively with the number of pulmonary infections (*p* < 0.031, r = 0.7) (4). Conclusions: Cardiopulmonary comorbidities are a main factor associated with increased mortality in patients with AS, especially in hospitalized patients. The mortality rates among patients with AS admitted to hospital due to other conditions other than movement disorders exceed the populational risk. The number of biologically treated patients correlated negatively with hospital admissions due to IHD.

## 1. Introduction

Ankylosing spondylitis (AS) is an autoinflammatory disease affecting the spine and sacroiliac joints that is also associated with increased occurrence of comorbidities [1]. Comorbidities in spondyloarthritis (SpA) add to the burden of disease by contributing to disease activity, functional and work disability, and a higher risk for depression [2]. This leads to lower life quality [2] and higher overall mortality rates [3]. Thus, awareness of comorbidities in SpA is crucial to improve their screening and management of SpA [3]. Yet, due to rapid development of medical knowledge and implementation of new treatment methods, a shift in comorbidities and mortality in the general population has been observed [4], which also affects patients with autoinflammatory diseases [5]. Additionally, a rapid increase of socioeconomic costs of health care systems has been observed [6]; therefore, big-data, population-based research is required for clinicians and health care providers to focus their efforts to balance the cost-effectiveness. Cardiovascular diseases (CVD) were identified as the leading cause of death in patients with AS. Several studies showed that systemic inflammation as well as secondary lipid profile alterations lead to increased CV incidents [7]. Research on CVD and CVD-related death in SpA suggests a cause-and-effect relationship. Cohort and populational studies suggest that the prevalence of ischemic heart disease and cardiac failure is higher among patients with AS in comparison to the general population [7]. Moreover, despite a world trend of decrease in CVD-related mortality and disability, prognosis for AS patients is still less favorable. Gastrointestinal tract involvement is considered as one of the major comorbidities in AS. Occurrence of peptic ulcers, infection of Helicobacter pylori, and post-NSAID enteropathy have been widely described in AS. Inflammatory bowel diseases (IBD) are a group of chronic autoinflammatory diseases sharing their pathophysiology and pathogenesis with Spondyloarthropaties [8]. The clinical manifestations of ulcerative colitis (UC) mainly involve abdominal pain, diarrhea, and hematochezia [9]. According to available studies, in about 6% of patients with AS, IBD is being reported [10]. Also, several studies on comorbidities in AS have reported subclinical IBDs [11]. Interestingly, the occurrence of radiological signs of AS, without inflammatory back pain among patients with IBDs has been reportedly approximately in 3–5% of patients [11]. Occurrence of eye inflammatory diseases is considered as one of the major health problems because sight deterioration or loss of vision leads to the major disability and lower quality of life. Ocular involvement is frequently encountered in SpA, of which uveitis is the most common manifestation. Acute anterior uveitis (AAV) is reported in almost 40% of the patients with SpA [12]. Ocular manifestations in AS were widely addressed in population studies. As novel research has suggested, the occurrence of AAV, episcleritis, and scleritis is associated with unfavorable disease progression [12]. Also, some researchers suggest that the occurrence of AAV is associated with a higher CVD risk, yet this is still under research [13]. Constant inflammatory state and gene changes that lead to ankylosing spondylitis could also play a role in cancer development. The results from national registers [14,15,16,17] revealed that the overall incidence of cancer seems to be elevated in patients with AS, yet the outcomes between populations seem to differ. In patients from Hong Kong, Chang et al. [14] reported an increased risk of hematological malignancy in both sexes, colon cancer in females, and bone and prostate cancer in males. On the contrary, Feltelius et al. [15], in their retrospective analysis, reported only increased prevalence of kidney neoplasm, which was not observed by Bittar et al. [16]. Research by Bitter stated that among patients with AS, skin cancers (squamous cell, malignant melanoma, and basal cell) and head and neck cancers were reported as significantly increased [16]. Similar observations were made by Kagan et al. [17], who reported increased risk for solid tumors in AS.

Accurate estimates of treatment trends and populational comorbidities drifts are important in planning health policy [18,19]. Data obtained from outpatient clinics are often lacking due to the requirement of reporting a single ICD-10 number. We decided to analyze data acquired from the General Hospital Morbidity Study conducted by the Institute of Public Health—National Institute of Hygiene (NIH-PIB), as health care providers, except psychiatric facilities, are required to send data to the NIH-PIB. Because AS affects less than 0.5% of the global population, observational or single-center studies on small groups do not provide enough data for estimating population drifts or trends in comorbidities or mortality. There is a need for studies performed on large populations. Yet, due to COVID-19 epidemics, data from years 2021–2023 are burdened with the influence of a strong single factor dominating general mortality rates. This also includes populational studies on mortality and comorbidities in AS. Therefore, we decided to analyze pre-pandemic data from the years 2011–2020 involving over 53,000 hospitalizations. In our study, we wanted to present trends and changes of comorbidities with concomitant mortality in patients with ankylosing spondylitis in Poland.

## 2. Materials and Methods

Raw data were acquired from National Institute of Public Health—National Institute of Hygiene (NIH-PIB) as anonymized numerical data from the 2011–2020 period. Data consisted of up to four ICD-10 codes with patients’ unique number, age, and additional procedures presented with ICD-9 codes. Each study procedure complied with the Declaration of Helsinki or similar standards of ethics. All patients’ data were searched for relevant diagnosis codes for AS.

### 2.1. Study Population

The study was approved by the local ethical committee (KBT-2/3/2022). Data were obtained from the NIH-PIB database, regarding patients diagnosed with AS (M45) from 1 January 2011 to 31 December 2020 covering 95% of the Polish population. The NIH-PIB collects information on patients including sociodemographic data, ICD-10 coded diagnoses, procedures patients underwent as ICD-9 codes, dates of admission and discharge, sex, and cause of death. The authors excluded repeated hospitalizations from analysis.

We used the following criteria for inclusion in this analysis: (1) diagnosis of M45, and (2) age above 18 years (3). We excluded patients who (1) did not meet these criteria and (2) were diagnosed with other autoinflammatory disease or autoimmune disease including M00, M05, M06, M46, M31, M32, M33, M35, and M31. Patients diagnosed with L40 (psoriasis), sarcoidosis (D86), reactive arthritis (M02), gout (M10), and infective arthritis (M00) were excluded from the study. Patients diagnosed with overlapping inflammatory bowel diseases (K50) were included in the analysis if they were also diagnosed with M45. Patients reported with an ICD-9 code of 00.181 or 00.18 (Introduction of Therapeutic Monoclonal Antibody) were considered as biologically treated. In the study group, we defined and analyzed a group with ischemic heart disease (IHD)—patients with the presence of diseases identified by the ICD 10 I20.0–ICD 25.9

### 2.2. Statistical Analysis

Statistical analysis was performed with the PS IMAGO v 26 SPSS module. Data were expressed as mean (±SD) for normal distribution and as interquartile ranges (IQR) for non-normal distribution. A Shapiro–Wilk test for normality for all measured parameters was performed. Pearson’s test for normal ranges and Spearman’s correlation test for not normal ranges were performed. For comparing non-paired nominal data, we used the Chi2 and Wilcoxon tests. A McNemar test was used for paired numerical data. A 2-sided *p* < 0.05 or an IRR whose 95% CI excluded 1.0 was considered statistically significant.

## 3. Results

Into our analysis, we included N = 53,142 hospitalizations (males N = 38,974 vs. females N = 14,168 *p* < 0.001), with a median age of 46.6 ± 13.7 for males and 48.5 ± 13.5 for females (*p* = 0.001) (Table 1). Statistically significant differences in reported comorbidities were found predominantly in the male population in comparison to female patients in cardiovascular diseases (n = 6546 patients) (2.7% vs. 1.3% *p* < 0.001) and pulmonary conditions (1.2% vs. 0.8% *p* < 0.023) ratios. After analysis of the correlation matrix, we found positive correlations between CVD and pulmonary conditions (*p* < 0.031 tau = 0.5). After regression analysis, we concluded that they are secondary to the “suppressor effect” based on collinearity (VIF = 14; tolerance = 0.1) between pulmonary diseases, such as COPD, pulmonary infections, and cardiovascular diseases in male patients. In the other groups, no statistically significant differences were found. Among CVD diseases, the most common was hypertension (HT) affecting 15% of patients, yet after an adjustment for age above 40 years, the percentage rose up to 25%. In hospitalized patients, group CVD was present in 45% of inpatients. Similarly, among the pulmonary comorbidities of hospitalized patients, the most common were infections (22%) and COPD/Asthma (32%). There were no significant differences in the occurrence of neoplasmatic or hematological diseases, traumas, and kidney diseases. The general mortality was 2.5%, yet analysis of subgroups of patients admitted to hospital due to conditions other than AS revealed a higher mortality of 13%. In Table 1, we present the typical clinical features of AS reported by medical services providers. In the statistical analysis, occurrences of acute anterior uveitis were not statistically significant between the male and female patients (1.7% vs. 1.6% *p* < 0.34). In the female group, we observed a tendency toward a higher occurrence of inflammatory bowel diseases (2.3% vs. 1.7 *p* < 0.031) despite a similar ratio of ICD10-ths codes reported involving ICD-10:K symbol (gastrointestinal conditions). There were no significant differences in skin conditions other than psoriasis and osteoporosis.

In the further analysis, we included patients admitted to the hospital with a reported code for AS (M45) between the years 2011–2020. Data were divided by categories following the ICD-10 codes, as shown in Table 2. Groups of ICD-10 including P00–P96 (certain conditions originating in the perinatal period), Q00–Q99 (congenital malformations, deformations, and chromosomal abnormalities), V01–Y98 (external causes of morbidity and mortality), H60–H95 (diseases of the ear and mastoid process) were excluded from analysis because their frequency was too low for the requirements of the statistical analysis.

### 3.1. Hospitalisations Due to AS

In the analysis of all hospitalizations, a decrease in number of hospitalizations in patients due to AS was observed. Also, a decline in the total number of hospitalizations (*p* < 0.031), hospitalizations due to cardiovascular diseases (ICD-10: I; *p* < 0.041), and movement disorders (ICD-10: M; *p* < 0.051) were observed. There were no statistically significant differences in hospital admissions due to neoplastic processes (ICD-10: C, D and Z), kidney diseases (ICD-10 N), infections (ICD-10: A, B, J and U), or trauma (ICD-10: T, S). Interestingly, despite a numerical decline, differences in the proportions presented in the percentages between the analyzed conditions remained similar and statistically insignificant, as shown in Table 2.

### 3.2. Overall Hospitalization and Comorbidities Risk Analysis

**Figure 1:**
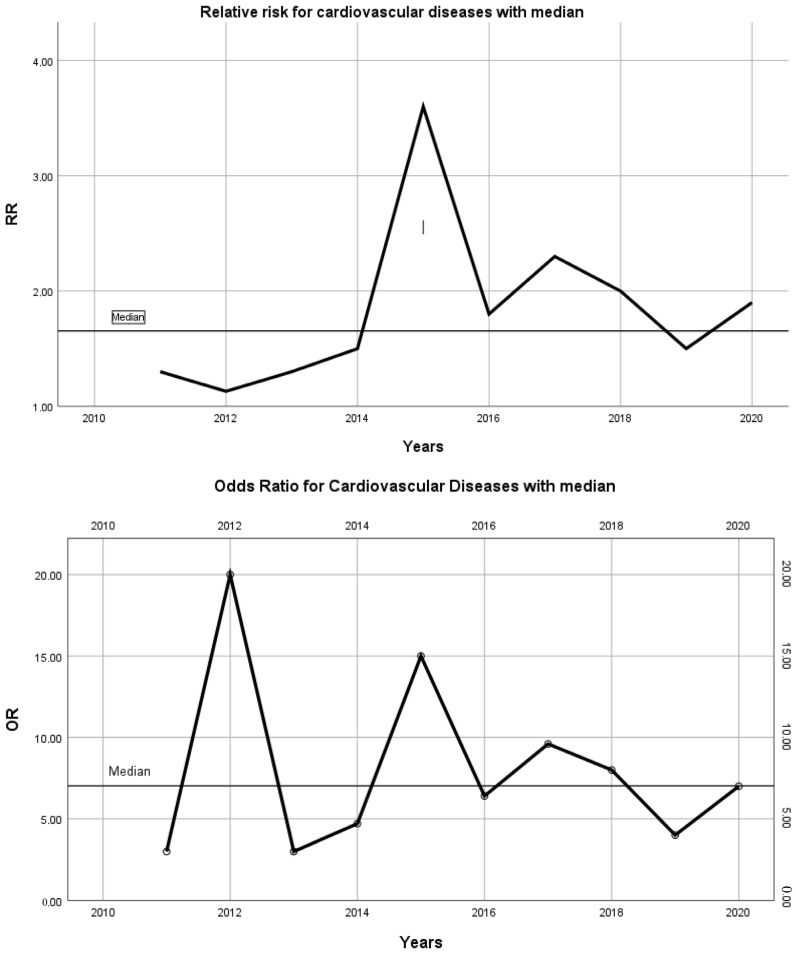
Relative risk (RR) ratios and odds ratio (OR) changes for cardiovascular conditions in patients with AS with medians.

On the trend analysis, we calculated relative risk (RR) ratios of the mortality for statistically significant (*p* < 0.05) factors based on Table 2. In Figure 1, we present the changes in RR with the time trends for CVD (Figure 1) and for pulmonary conditions. As shown on Figure 1, there is a tendency toward increased mortality among patients with AS who were hospitalized (RR median: 1.6 OR median: 7). Despite, the observed lowering in occurrence of CVD, the relative risk ratios remained on a stable level. Similarly, the CVD pulmonary conditions presented similar tendencies with increased mortality rates (RR median: 2 OR median: 5) and similar mortality risks despite the observed trends.

**Figure 2:**
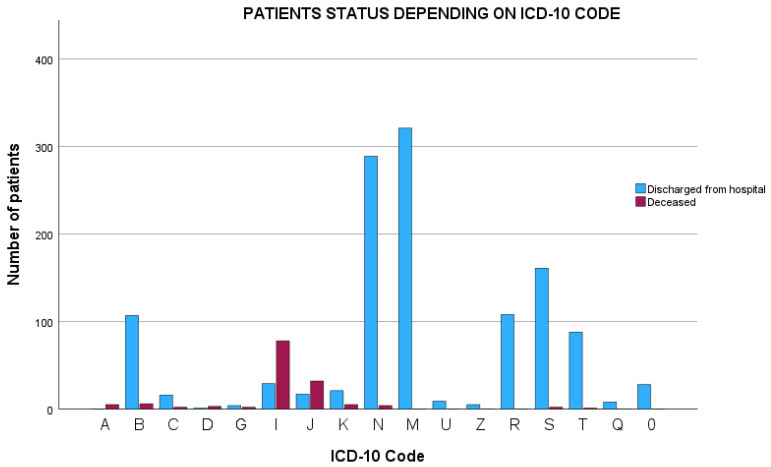
Percentage share of ICD-10 related deaths.

In the further analysis, we included only patients with AS admitted to hospitals due to conditions other than M45. In the analysis, we included n = 1562 patients and among them 156 deaths were reported. We analyzed direct, secondary, and underlying causes of death. Ambiguous codes such as R09.2 (lack of breath) that didn’t refer to a direct cause of death were excluded unless an unambiguous secondary or underlying cause was given. Cases without secondary or underlying or other following conditions were excluded from our analysis. In the statistical analysis, we used the Chi2 test and multivariate logistic regression analysis. In the logistic regression, we created a statistically significant model including two covariates (chi-square *p* < 0.001, Hosner–Lewershaw test *p* = 0.31, Nagerkelke R Square = 0.38) with two covariates: cardiovascular diseases (B = 5.7 *p* < 0.01) and pulmonary conditions (B = 3.0, *p* < 0.031), as presented in Figure 2 (ICD-10: J and I). Further analysis suggests a strong collinearity (VIF > 4; tolerance < 0.25; condition index > 30) between those two variables. No statistical significance was observed for the other analyzed factors.

### 3.3. Associations between Initiation of Biological Treatment and Comorbidities

**Figure 3:**
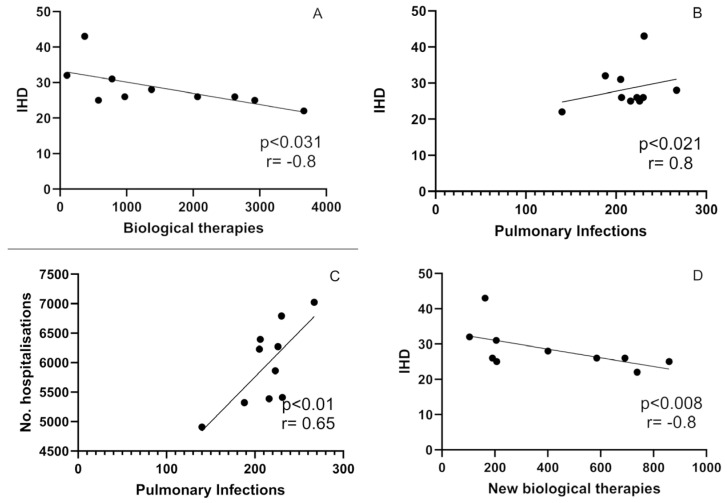
Correlations between (**A**)-cardiovascular diseases and number of biologically treated patients (**B**)-Correlations between IHD and pulmonary infections (**C**)-orrelation between hospitalization and pulmonary infections (**D**)-Correlations between IHD and new biological treatment programs.

A total number of 4056 patients (7%) were hospitalized to administer the dosage of biological treatment (ICD-9 code 00.181). Demographic data are given in Table 3. In Poland, biological treatment is carried out in accordance with the EULAR standards [19], taking into account the recommendations of the Polish Society of Rheumatology and Polish law regulations. Patients with severe infection and pregnant were omitted from the analysis. Patients who qualified for biological therapy had lower rates of comorbidities, which can be explained partially by contradictions to anticytokine treatment, and so this that analysis was limited. Occurrence of uveitis was higher in the biologically treated group in comparison to the remaining patients with AS (10% vs. 3%). Since 2016, we observed a significant rise in the number of patients qualified for the biological treatment, namely from 2% in 2011 to 16% in 2020. We further analyzed the associations between comorbidities and hospital admission between 2011 and 2020, and significant correlations between the measured parameters, shown in Figure 3A–D. The total number of n = 256 patients were admitted to the hospital due to IHD. We found a statistically significant negative correlation between the number of patients with IHD per year and the number of patients initiating biological treatment (*p* < 0.031, r = −0.8), shown in Figure 3A. Also, the number of hospital admissions due to IHD correlated strongly and positively with the number of patients with pulmonary infections (*p* < 0.021 r = 0.8), given in Figure 3B. There was also a significant positive correlation between the total hospitalization count and pulmonary infections (r = 0.65; *p* < 0.01), as shown in Figure 3C. Finally, we observed a strong negative correlation between the number of IHD-based hospitalizations and the number of newly biologically treated patients per year (r = −0.8; *p* < 0.008), Figure 3D. There were no statistically significant correlations between the number of biologically treated patients and the number of newly diagnosed patients with neoplasms, hematological malignancies, and skin conditions, other than AS movement disorders, endocrinological disorders, eye disorders, neuropsychiatric conditions, or other conditions during pregnancy. There was only a statistically significant decline in the occurrence of CVD (*p* < 0.001) and pulmonary conditions (*p* < 0.001), but changes in the remaining comorbidities were statistically nonsignificant. There were also no correlations between the number of patients initiating biological treatment and hospital admissions or with overall hospital mortality.

## 4. Discussion

Increasing comorbidity, mortality, and associated socioeconomic costs position AS as a major public health problem. Patients with AS are burdened with higher cardiovascular risk as compared to the general population [20,21]. Following Gökşenoğlu et al. [22], the population with AS is burdened with more comorbidities that also seem to affect predicted pharmacological and surgical treatment outcomes [23]. Interestingly, despite differences between the enrolled populations in terms of chronic kidney disease or diabetes [22,24], our data are consistent with other research showing that AS is not associated with an increased risk of cancer or hematological malignancies [24,25]. Our first and key finding is that, despite the decline in hospitalization rates, relative mortality ratios from cardiovascular (CV) and pulmonary conditions remain higher among patients with AS compared to the observed for the general population admitted to the hospital. This is especially highlighted in male patients with AS. According to population studies, cardiovascular and circulatory system involvement is the main cause of an increased risk of stroke, myocardial infarct, and sudden cardiac death [21]. The increase in the general death ratio in the AS population is estimated from 1.36-fold up to 2.3 times [3,26] with a 50% higher cardiovascular risk (CVR) [26] in comparison to the general population. This phenomenon seems to originate from two main sources. First are the populational factors from INTERHEART [27] study such as smoking, gender, genetic factors, and lipid profile. Second are the factors that are secondary to chronic inflammation, typical for SpA [28,29,30]. So far, several studies have investigated factors suspected of being associated with an increased cardiovascular risk and with disease activity in patients with AS [31,32,33]. Also, there were data suggesting that clinical symptoms such as uveitis were associated with increased CVR [12,13]. Despite the high percentage of patients treated for uveitis in our study, we did not observe significantly higher mortality nor CV conditions in this group of patients. Also, in our study, there was no significant correlation between the occurrence of uveitis and the risk for mortality or other comorbidities. On the contrary, populational studies from Hong Kong by Feng et al. [34] had shown that uveitis is associated with a higher risk for mortality and risk for developing IHD. There are several differences between our study and the one conducted by Feng et al., as our study was based on the ICD codes, while the work by those authors was based on clinical data. Although there were more patients with AS included in our research, the group treated due to uveitis was relatively smaller (1400/53,145 vs. 1111/5555). Also, due to the retrospective nature of this study, our observations should be treated with caution.

Despite the limitations of our study, overall cardiovascular mortality in our research group was a total of 2.5% for all AS patients and 13% for patients with AS who were hospitalized for comorbidities other than movement disorders. Although the general mortality of the AS patients enrolled into our study surpassed 0.5% of the mortality described for the European population [35], our data were acquired from hospitals and are not comparable with those for the general population.Therefore, they should be addressed in a separate study. Yet, despite the limitations of this study, mortality due to comorbidities among in-hospitalized patients with AS(13%) was higher than one described by Walicka et al. for average mortality (4.1%), as well as for short term (5–7 days long) hospitalizations (2.63%) in Poland [36]. Also, in our cohort, CVD-related mortality (13% vs. 11.4%) as well as mortality related to pulmonary diseases (13% vs. 7.4%) was higher than the estimates for the general Polish population [37]. Yet, again due to the selective nature of our study, such observations should be verified in a separate study.

The EUROASPIRE III and EUROASPIRE IV (European Action on Secondary Prevention by Intervention to Reduce Events) survey showed that hypertension (HT) is the main cardiovascular risk factor [38,39], which translates to 40.6% of deaths from cardiovascular disease globally [40]. Our research shows that HT affected 15% of patients diagnosed with AS, which was compatible with populational studies [40,41,42,43] and similar to the research in the general Polish population by Liput-Sikora [44]. Yet, in our research after adjustment for age above 40 years, this percentage rose up to 25% in hospitalized patients. Such a discrepancy matches the populational data from the general population studies such as the NATPOL 2011 Survey (22%) and WOBASZ I and WOBASZ II (33%) [43]. Also, similar trends were described for the general Polish population [43]. Yet, despite positive trends for HT, mortality ratios and relative risk remain high despite the general trend for lowering of cardiovascular mortality in Poland [45]. This observation suggests discrepancies between patients with AS and the general population, supposedly delivering from chronic inflammation and disease activity. Yet, our data represent trends for the mainly male, high-CV risk population. Because of typical for AS imbalance in the male/female ratio, the presented data do not fully relate to the general population and should be interpreted within the context of studies focusing on AS.

Consequently, our findings show associations between a risk of hospitalization and cardiopulmonary comorbidities. Our outcomes stay in line with other research such as the study by Bittnar et al., which was obtained from the US population [16] and other studies [46]. In the analysis of hospitalized patients, we found that 45% of patients were diagnosed during hospitalization for CV condition, but also that 32% of AS patients were hospitalized due to obstructive airways diseases. Also, among inpatients, 22% were admitted to the hospital due to respiratory tract infections. This observation seems to originate from multiple overlapping factors. Lung diseases are associated with environmental factors such as air pollution [47,48], cigarette smoking, and several other risk factors. In recent years, research has focused on phenotypes of lung changes observed in the course of ankylosing spondylitis. Despite the fact that UIP/NSIP is not commonly associated with AS [49], several researchers had reported higher rates of pleural thickening, fibrotic changes, emphysema, and non-specific interstitial changes compared to the healthy controls [47]. Impairment in lung function leads to increased vulnerability for infections and development of COPD/emphysema, especially in actively smoking patients [48]. Also, the aforementioned conditions tend to share similar risk factors with secondary pulmonary hypertension, heart valvular diseases, arrhythmias, and chronic heart failure [49], further increasing the overall CV risk and general mortality rates. This issue has been addressed by Shrikrishina et al. in the European Heart Journal [43], where the authors suggested a new term, namely a cardiopulmonary risk. The above observations stand in line with the others available, supporting our findings considering the collinearity between CVD and pulmonary conditions [43]. Also, the rapidly increasing number of biologically treated patients seem to correlate negatively with the number of patients with AS admitted to the hospital with IHD. Yet, despite the promising results, due to the retrospective nature of this register analysis and the lack of clinical data (such as traditional CV risk factors) on biological treatment, we cannot directly associate the initiation of biological treatment with a decrease in IHDs, and so this should be addressed in further studies. Due to the small number of patients diagnosed with AS (below 0.5% of population) and overlapping multiple factors, the research on the populational scale is difficult in AS. Considering the limitations of our study, the most efficient further approach would be combining populational and clinical data in the form of a register, such as the British Society for Rheumatology Biologics Register in Ankylosing Spondylitis (BSRBR-AS) [50] or the Framingham Heart Study [51].

Our second finding concerns the different disease phenotype in female patients with AS. Our study revealed a higher occurrence of inflammatory bowel diseases in female patients. Several other researchers [50,51,52] had reported similar clinical differences in the SpA’s disease progression, but also higher rates of subclinical bowel inflammation in female patients with AS [8,53,54]. In other studies, higher ratios of insidious back pain, worse responses to anti-TNF treatment, and higher rates of peripheral joint inflammation [52] were described in females with AS. Therefore, a personalized clinical approach should be advised, considering other than axial symptoms of SpA in female patietns.

Finally, our study has several limitations. First, our analysis was retrospectively based on reported ICD-10 codes and in-hospital procedures and not on real life data. This analytic approach limits our outcomes because we were unable to analyze clinical data, biochemical parameters, or potential differences between biological drugs used in treatment of AS. Secondly, due to our retrospective approach, we were not able to verify the diagnosis of AS/M45 or obtain additional data on disease progression nor clinical data such as occurrence of dactylitis, spondylitis, or other comorbidities such as dyslipidemia or obesity. Also, we encountered difficulties during analysis that are typical for register-based studies, mainly the lack of data and necessity to exclude missing valuables from analysis. Finally, we were unable to perform a direct cause–effect analysis of multiple overlapping factors such as biological treatments on CV risk or comorbidities due to the lack of clinical data.

## 5. Conclusions

A decrease in hospitalizations of patients with AS was observed, yet overall mortality in the AS population seems to be higher than in the general Polish population. Despite a decrease in hospitalization rates and morbidity remaining at a stable 2.5% level, in-hospital mortality exceeded the indices for the general population in patients with AS when they were hospitalized due to comorbidities. Cardiopulmonary comorbidities seem to be a main factor associated with increased mortality rates and higher than populational mortality. Our research shows that biological treatment might be associated with lower risk of IHD, yet due to the limitations of our study it should be approached with caution and addressed in further research. Male patients with AS seem to be at a higher risk of overall mortality; thus, prevention and health programs should focused on patients with AS.

## Figures and Tables

**Table 1:** Clinical and demographic data comparing male and female groups. Data are presented as means ± SD or in percentages.

	All Patients	Males	Females	*p*
Age (years)	47.1 ± 13.7	46.6 ± 13.7	48.5 ± 13.5	<0.001
Gender(Male/Female)	53,142	38,974	14,168	<0.001
Median length of hospitalization (Days)	2 [0–10]	2 [0–10]	2 [0–9]	0.071
Psoriasis [%]	710	325 (45%)	389 (55%)	0.4
Eye inflammation [%]	882 (1.7%)	658 (1.7%)	224 (1.6%)	0.392
Inflammatory bowel diseases [%]	996 (1.9%)	675 (1.7%)	321 (2.3%)	<0.001
Crohns disease [%]	295 (0.6%)	157 (0.4%)	138 (1.0%)	<0.001
Colitis Ulcerosa [%]	702 (1.3%)	519 (1.8%)	183 (1.3%)	0.721

**Table 2:** Hospital admissions divided by year of admission and ICD-10 codes.

	ICD-Code	2011	2012	2013	2014	2015	2016	2017	2018	2019	2020
Certain infectious and parasitic diseases	A00–B99	40	52	40	51	57	52	47	49	49	39
Neoplasms	C00–D49	42	54	55	84	73	102	104	117	113	95
Diseases of the blood and blood-forming organs and certain disorders involving the immune mechanism	D50–D89	93	83	91	73	99	83	72	130	82	102
Endocrine, nutritional, and metabolic diseases	E00–E90	389	395	461	454	595	615	498	604	604	470
Mental and behavioral disorders	F00–F99	41	41	27	29	33	27	19	50	41	31
Diseases of the nervous system	G00–G99	81	73	88	92	129	111	157	80	94	81
Diseases of the eye and adnexa	H00–H59	76	164	220	220	223	209	92	110	108	88
Diseases of the circulatory system	I00–I99	999	1155	1102	1254	1317	1294	1118	1100	1128	802
Diseases of the respiratory system	J00–J99	188	231	216	205	230	267	206	227	226	140
Diseases of the digestive system	K00–K93	232	284	363	355	388	387	322	441	413	384
Diseases of the skin and subcutaneous tissue	L00–L99	51	67	61	66	59	75	62	110	105	54
Diseases of the musculoskeletal system and connective tissue other than ankylosing spondylitis	M00–M99 without M45	927	1050	1199	570	3186	1307	1403	1230	1362	970
Diseases of the genitourinary system	N00–N99	165	156	206	177	184	203	208	189	207	110
Pregnancy, childbirth, and the puerperium	O00–O99	121	116	101	138	135	138	170	170	177	153
Symptoms, signs, and abnormal clinical and laboratory findings, not elsewhere classified	R00–R99	41	53	32	68	56	59	84	88	95	77
Injury, poisoning, and certain other consequences of external cause	S00–T98	92	80	68	86	83	85	100	85	90	93
Factors influencing health status and contact with health services	Z00–Z99	87	75	96	119	122	169	215	150	154	137

**Table 3:** Comparison of comorbidities in patients treated with biologics in comparison to non-biological treatment group.

	ICD-Code	Biological Treatment (n = 4056)	Non-Biological Treatment (n = 49,086)
Endocrine, nutritional, and metabolic diseases	E00–E90	15 (0.3%)	4481 (0.8%)
Mental and behavioral disorders	F00–F99	10 (0.2%)	298 (0.5%)
Diseases of the nervous system	G00–G99	5 (0.1%)	906 (2%)
Diseases of the eye and adnexa	H00–H59	400 (10%)	1400 (3%)
Diseases of the circulatory system	I00–I99	52 (1,2%)	10,169 (19%)
Diseases of the respiratory system	J00–J99	4 (0.1%)	1909 (4%)
Diseases of the digestive system	K00–K93	123 (3%)	3128 (6%)
Diseases of the skin and subcutaneous tissue	L00–L99	3 (0.007%)	600 (1%)
Diseases of the genitourinary system	N00–N99	10 (0.02%)	1616 (3%)

## Data Availability

Data are available upon reasonable request according to ICMJE requirements: Data shared: All the individual participant data were collected during the trial, after deidentification. Available documents: study protocol, statistical analysis, analytic code. When will data be available: beginning three months and ending two years following the article’s publication. With whom: Researchers who provide a methodologically sound proposal. For what type of analyses: to achieve the aim of the approved proposal. By what mechanism will data be made available: The proposal should be directed to katarzyna.helon@spartanska.pl.
